# An Historical Perspective on How Advances in Microscopic Imaging Contributed to Understanding the *Leishmania* Spp. and *Trypanosoma cruzi* Host-Parasite Relationship

**DOI:** 10.1155/2014/565291

**Published:** 2014-04-27

**Authors:** P. T. V. Florentino, F. Real, A. Bonfim-Melo, C. M. Orikaza, E. R. Ferreira, C. C. Pessoa, B. R. Lima, G. R. S. Sasso, R. A. Mortara

**Affiliations:** Departamento de Microbiologia, Imunologia e Parasitologia, Escola Paulista de Medicina, UNIFESP, Rua Botucatu 862, 6th Floor, 04023-062 São Paulo, SP, Brazil

## Abstract

The literature has identified complex aspects of intracellular host-parasite relationships, which require systematic, nonreductionist approaches and spatial/temporal information. Increasing and integrating temporal and spatial dimensions in host cell imaging have contributed to elucidating several conceptual gaps in the biology of intracellular parasites. To access and investigate complex and emergent dynamic events, it is mandatory to follow them in the context of living cells and organs, constructing scientific images with integrated high quality spatiotemporal data. This review discusses examples of how advances in microscopy have challenged established conceptual models of the intracellular life cycles of *Leishmania* spp. and *Trypanosoma cruzi* protozoan parasites.

## 1. Introduction

**Figure 1:**
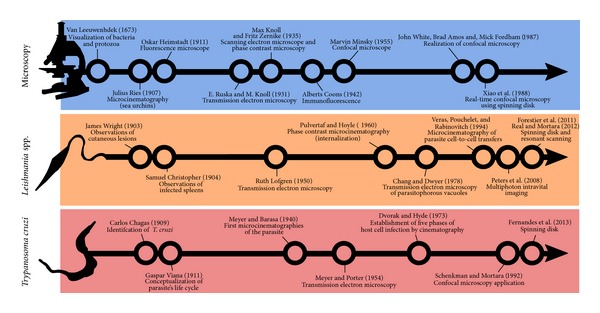
Timeline showing important historical achievements in microscope technology and* Leishmania *spp./*T. cruzi *knowledge. References from the timeline are shown in the text, and additional references are cited in the figure [10, 11], revised in [12, 13].

Leishmaniasis and Chagas disease are tropical diseases caused by protozoan parasites from the Trypanosomatidae family (*Leishmania* spp. and* Trypanosoma cruzi*, resp.). These protozoans belong to the class Kinetoplastea, a group of flagellated organisms with a peculiar organelle called a kinetoplast and a single mitochondrion [1]. These two trypanosomatids are responsible for approximately 20 million reported cases of leishmaniasis and Chagas disease and 100,000 deaths per year, primarily in tropical and subtropical areas of the globe [2]. The negative economic and social impact of these diseases, especially in Central and South America, is of great concern [3] and has stimulated scientific investments into studying their causative agents. Because the pathogenesis of* Leishmania* spp. and* T. cruzi* involves an intracellular life cycle in human and mammalian hosts, interactions between the parasite and host cells have been extensively studied* in vitro*, with particular emphasis on microscopic observations. A timeline showing important historical achievements in microscope technology and* Leishmania *spp./*T. cruzi *knowledge is presented in Figure 1.

Remarkable technological advances have increased our ability to sense or experience microscopic agents, building concepts from scientific images. Researchers “embody” technology, boosting his/her experience: scientific images are obtained after technological mediation between researchers sensorial apparatus (perception) and the object of study [4]. Increased spatial resolution with the advent of electron microscopy (EM) enabled access to high quality spatial data for studying the relationship between host cells and pathogens. EM was, and still is, extremely important in determining how viruses, bacteria, fungi, and protozoan parasites (such as* Leishmania* spp. and* T. cruzi*) interact with host cells. However, the singularity of temporal data and lack of integration between high spatial resolution and access to the same individual at different time points (due to chemical fixation of samples) led to a fragmented experience of the object and, unfortunately, limitations in a full understanding of how parasites establish and propagate themselves within their hosts (Box 1).

Factual statements (singular propositions) fragmented in space and time can produce temporal, spatial, and causal gaps in experiences, which may be solved by constructing conceptual models using solid statistical historical fundamental principles. Due to partial agreement with nature, models have an important predictive power (although to a limited extent) in building an interpretative framework for other researchers until new information (obtained after technological improvements) challenges and rebuilds these frameworks [9]. The life cycles of protozoan parasites, from invasion and colonization to spreading within the host, are conceptual models based primarily on a reductionist approach that considers nonintegrated time and space observations.

Live recordings of the host-pathogen relationship have been produced as microcinematographic and video technology has progressed, but the large majority of these studies lack appropriate spatial resolution to observe detailed aspects of the interaction. Integrated or four-dimensional observation of objects approximates our experience to microscopic dynamic states, such as oscillatory or chaotic behavior, that are unreachable under the conceptual frameworks of static stability and conventional imaging technology, fixed at defined time points or contained in limited spatial/topological regions of the sample [8].

Herein we use* Leishmania *spp. and* T. cruzi* as examples of how advanced microscopic techniques are circumventing reductionism, integrating or reaching further dimensional scales, and unveiling new aspects of host cell-parasite relationships. Observations of these protozoan parasites will be discussed from a historical point of view considering breakthrough studies and acquisition of new information based on integrated spatiotemporal data.

## 2. Imaging* Leishmania* spp. and Host Cells

In 1881, Alphonse Laveran (1845–1922) found that a protozoan was the etiological agent that caused malaria, which encouraged researchers in the field of protozoology to describe and investigate protozoan pathogens transmitted to human hosts, especially those carried by insect vectors. This conjuncture led to the investigation of an ancient human malady described in diverse manners in antiquity and modern times [22, 23]. Discovery of the etiological agent that causes leishmaniasis, a protozoan parasite from the Trypanosomatidae family, and conceptualization of its life cycle were established from key observations in accordance with Koch's postulates and paradigms of infection and pathogenesis: identify and isolate the microorganisms, cultivate them* in vitro, *and establish a causal relationship with disease. In microscopic observations of Delhi boils, Scottish Surgeon Major David Douglas Cunningham (1843–1914) found a round-shaped parasite inhabiting cells, and Piotr Borovsky (1898), who observed similar skin lesions (Sart Sore, Turkmenistan), suggested that the intracellular bodies were protozoans. William Leishman (1865–1926) and Charles Donovan (1863–1951) found similar organisms in tissues extracted from the viscera of fatal cases of kala-azar in India. Attempting to cultivate these organisms* in vitro*, Leonard Rogers (1868–1962) and Charles Nicolle (1866–1936) extracted the round-shaped protozoans from infected tissues and cultivated them in blood agar culture media. Multiplying flagellated protozoan forms were found in the culture medium, which led to the conclusion that the parasite was a trypanosomatid. Edmond Sergent (1876–1969) and colleagues found that trypanosomatids could be digenetic parasites, transmitted from insects to mammals [24], and suggested the same life cycle for those protozoans, which were then classified as* Leishmania*.* In vitro *cultivation of these parasites allowed their inoculation into dogs, monkeys, and small rodents, which subsequently developed pathologies similar to the human disease. In 1921, it was experimentally demonstrated that* Phlebotomus*, a tiny sand fly, is the insect host for* Leishmania* and the transmitter of leishmaniasis [24, 25].

Wright (1869–1928) in 1903 [26] and Christophers (1873–1978) in 1904 [27] observed that cutaneous lesions or infected spleens presented massive infiltration of cells containing a large number of oval-shaped parasites. Christophers was the first to recognize these preferentially infected cells as macrophages, inferring that phagocytosis was responsible for the uptake of parasites by leucocytes [26, 27]. For decades, leishmaniasis was considered a disease almost exclusively of the host macrophage system [28], and phagocytosis is still considered the primary mechanism of* Leishmania* spp. internalization [29].

Pulvertaft and Hoyle [30], 56 years after Christopher's inferences, recorded the phagocytosis of* Leishmania* spp. by monocytes/macrophages. Using phase contrast live microcinematography, the authors described monocyte pseudopodia reaching and taking up leptomonad forms (now generally called promastigotes) of* L. donovani*. The promastigotes display a single flagellum in their anterior poles; Pulvertaft and Hoyle demonstrated that promastigote phagocytosis took place from the opposite pole, the posterior, within several minutes. After total engulfment, a vacuole is observed around the parasite that may be digested and disappear or, alternatively, survives and remains motile within this compartment. However, Miller and Twohy (1967) [31] and Akiyama and Haight (1971) [32] found that hamster macrophage pseudopodia initially internalized promastigotes by the flagellar anterior pole of the parasite and observed a transient vacuole around it.

Forty years later using 3D and 5D reconstruction images, Forestier and coworkers (2011) [33] observed that* L. donovani* promastigote uptake by macrophages occurs mainly by the flagellar tip and could also, in exceptional cases, occur through the posterior region and lateral portions of the body. The authors described four sequential phases of* L. donovani* promastigote establishment in host cells: (i) highly polarized attachment by the flagellar end and internalization in lysosomal compartments; (ii) reorientation; (iii) oscillating movement of the parasite to the periphery of the host cell associated with lysosome exocytosis and minor damage to the host cell; and (iv) loss of motility and final location of the parasite in parasitophorous vacuoles (PVs) near the host cell nucleus. These conclusions were only possible due to cutting-edge, high-speed live imaging under modern microscopes [34]. Courret and colleagues (2002) observed similar polarized entrance of* L. amazonensis* promastigotes into macrophages using conventional live imaging techniques of infected samples.

The investigation of* Leishmania* internalization by macrophages largely benefited from transmission electron microscopy (TEM). Host cell pseudopodia are formed around entering parasites with concomitant microfilament aggregation; sites of close contact between parasite and host cell membranes can be visualized in detail using this technique [35]. In 1986, Wozencraft and colleagues used EM to map individual molecules involved in* Leishmania*-macrophage interactions. Using immunogold labeling, complement receptors were observed to be associated with the interface between membranes of the macrophage and the interacting* Leishmania,* but not with internalized parasites. These observations confirmed results published in the same year, demonstrating participation of this receptor in the direct binding of macrophages to* Leishmania* promastigotes [36]. It is now recognized that* Leishmania* internalization by macrophages is tightly modulated by the first and third complement receptors (CR1 and CR3) and mannose (MR) and Fc gamma receptors (Fc*γ*R) [29].

*Leishmania* internalization by macrophages involves accumulation of actin filaments at the internalization sites of the parasite, a feature of phagocytosis [37]. The authors of the first studies on the mobilization of host cell components towards phagocytosed parasites benefited from immunolabeling techniques associated with electron and optical microscopy. The use of antibodies conjugated to fluorophores proved to be an easy, accessible technique to study protein distribution in cell biology [38]. Regarding* Leishmania* phagocytosis, fluorescence immunolabeling of host GTPases and actin labeling enabled the observation that these molecules are colocalized during* Leishmania*-macrophage interaction [39]. Further, the authors found that different GTPases, Rac1 and RhoA, regulate internalization of opsonized and nonopsonized* Leishmania* promastigotes, respectively. Using the same immunolabeling technique, they also observed that internalization of nonopsonized amastigotes is alternatively regulated by Rac1 but, in this case, the oxidative burst triggered by host phagocytosis is restrained [40]. Thus, different receptors (for opsonized or nonopsonized parasites) trigger different GTPases that modulate host cell responses to* Leishmania.*

After internalization by host cells,* Leishmania* parasites are lodged in PVs, in which they multiply as oval-shaped amastigotes. Electron micrographs of* Leishmania *PVs acquired by Alexander and Vickerman in 1975 and Chang and Dwyer in 1978 demonstrated the phagolysosome-like nature of the vacuoles developed by this parasite [41, 42]. By loading host cell phagolysosome vesicles with electron-dense compounds, these compounds were observed inside* Leishmania *PVs, suggesting that PVs fuse with late endosomes and secondary lysosomes. In the 1990s, a series of studies from Jean-Claude Antoine demonstrated that PVs acquire early endosome markers such as Rab5 and EEA-1 that are substituted by late endosome markers, such as Rab7, and glycoproteins associated with lysosomes [43]. The resulting parasite-containing compartment is a “mature” PV presenting several phagolysosome features [34, 43–46]. PVs develop different morphologies according to* Leishmania *species:* L. mexicana* and* L. amazonensis*, for example, present a spacious PV containing several amastigotes, while most species (*L. major*,* L. donovani, *and others) present a tight-fitting PV in which PV and parasite membranes are in contact [47, 48]. PV biogenesis is still poorly understood, mainly because the majority of studies have been performed in fixed cells using endosomal/lysosomal membrane markers.

Spinning disk technology for confocal laser scanning allowed observation of PV biogenesis in live samples from the very early moments of infection at the stage of parasite phagocytosis. Multidimensional images obtained from these techniques allowed for integration of four and even five dimensions (*x, y, z, *time, fluorescence) of living cells and tissues [49]. Lippuner and colleagues [50] were some of the first researchers to record PV biogenesis in live samples using GFP-tagged Rab5 proteins on cells hosting* L. mexicana*. The authors demonstrated that the parasite inhabits PVs in which Rab5 GTPases are rapidly excluded from the vacuolar membrane (compared with latex bead phagosomes). They also documented that a parasite surface component, lipophosphoglycan (LPG), implicated in delaying PV maturation in* L. donovani *[39] accelerated the exclusion of the Rab5 marker from PVs.

Benefitting from high resolution and speed, as well as the low photocytotoxicity of the technique, Forestier and colleagues and Real and Mortara [33, 48] observed the interaction of PVs with acidified compartments of host cells. They dyed vesicles with a lysosomotropic probe (Lysotracker) over time and observed how these labeled vesicles compose PVs. These acidic vesicles were located around internalized promastigotes minutes after interaction with host cells, suggesting that recently formed PVs promptly fuse with acidic compartments [33]. The biogenesis of spacious/communal PVs formed by* L. amazonensis *versus tight-fitting PVs formed by* L. major *could also be compared using the technique. The growth of spacious PVs was accessed in terms of volumetric data in that remodeling restores PV dimensions after these large structures fuse together [48]. The fission of* L. major* PVs during parasite intracellular multiplication was also observed for the first time using GFP-tagged LAMP and Rab7 proteins and multidimensional imaging techniques. Thus, the PV membrane could be visualized during amastigote multiplication, unveiling the dynamics of PV fission [48].

However, some aspects of the* Leishmania* life cycle, such as putative host cell collapse due to parasite growth and amastigote spreading to other cells and tissues that must occur in disease persistence, are far from being elucidated and are only hypothetically mentioned in the literature. Laser scanning and/or spinning disk confocal microscopy and intravital imaging techniques are promising tools for investigating these dynamic events. It is difficult to conceive approaches to evaluate* Leishmania* egress/reinfection when only taking into account static information from fixed samples.

Considering the seminal works on leishmaniasis from the early 20th century, the preferential, almost exclusive, presence of oval-shaped parasites inside host cells was intriguing and suggested that the parasite was extremely dependent on the intracellular environment. If few parasites could be found outside host cells, the question remained as to how they could spread to other cells and tissues and induce skin and organ lesions after an insect-vector bite.

In 1980, Dennis Snow Ridley, an expert in the pathology of leprosy, was one of the first to attempt to study* Leishmania *egress from a host cell [51]. In fixed histological samples from lesions, he observed “macrophage lysis and the presence of extracellular amastigotes in forms of disease in which parasite numbers were restricted, but not in those in which parasites were freely tolerated.”

In the late 1990s, Rittig et al. [52] used time-lapse microscopy of infected human peripheral blood monocytes to properly investigate the dynamic event of* Leishmania major *egress from host cells [52, 53]. They found “numerous host cells simultaneously releasing replicated parasites” in an exocytotic-like process. Also in the 1990s, a series of unpublished cinematographic records of macrophages hosting* L. amazonensis* was made by Michel Rabinovitch and collaborators at the Institut Pasteur in Paris, France. The recordings show transference of amastigotes from macrophage-to-macrophage and infected lymphocytes being phagocytosed by macrophages, similar to Trojan horses (supplementary Video 1). These time-lapse approaches challenged the current understanding of* Leishmania *egress based on bacterial and viral conceptual intracellular cycles, which presume host cell lysis by multiplication bursts [47].

Although still hypothetical, these egress events are crucial for* Leishmania *parasites to reach the preferential intracellular niche of macrophages after their inoculation site on the mammalian host skin. From the insect blood meal to establishment inside macrophages,* Leishmania *parasites are likely transferred from cell to cell, a process that involves diversified host cell lineages. After* L. major *promastigote forms were inoculated in mice by sand flies, an intense migration of neutrophils was observed at the site of an insect blood meal 40 minutes post-inoculum [54]. The work employed multiphoton intravital microscopy (MP-IVM) on mice ear sites where infected sand flies had their blood meal. The technique allowed access to information contained in high depth tissues during transfer of parasites from insects to mice. Neutrophil-depleted mice had a decreased number of parasites after one and four weeks of* Leishmania* inoculation in their ears. This suggests that neutrophils are essential partners in establishment of the parasite in mammalian hosts in the early stages of infection. Relocation of* L. major* parasites from neutrophil to macrophage populations was inferred after six days post-inoculum, suggesting a transit of parasites between these two cell types.

Using similar microscopy techniques, dendritic cells were included as* Leishmania *host cells involved in early establishment of the parasite in mammalian organisms [55]. Injection of* L. major *promastigotes into the dermis of mice expressing fluorescent-tagged dendritic cells revealed that these cells avidly internalize parasites in the first three hours post-inoculum.

Thus, neutrophils and dendritic cells could participate in* Leishmania *pathogenesis as transient hosts until the parasite reaches its preferential niche, the macrophage. In neutrophils,* L. donovani *promastigotes are sheltered in harmless, nondegradative vacuoles until host cell apoptosis. Similar to a Trojan horse, the apoptotic neutrophil is phagocytosed by macrophages that safely transfer the parasites without exposure to the potentially hostile extracellular milieu [56]. Another interesting tactic of* Leishmania* egress and transfer between host cells is mediated by host cell extrusions. As described by Rittig and Bogdan in 2000 [53], parasites are extruded from apoptotic host cells and immediately rescued by viable neighbor macrophages (manuscript in preparation).

## 3. Imaging* Trypanosoma cruzi* and Host Cells

In the early 20th century in Brazil, as* Leishmania *was being characterized in Europe, Carlos Chagas (1878–1934) identified the new protozoan* Trypanosoma cruzi, *its invertebrate host, and insect vector as well as pathological aspects. In 1909, Chagas named the protozoan* Schizotrypanum cruzi* as a tribute to Oswaldo Cruz, his director at Manguinhos Institute in Rio de Janeiro, Brazil [57]. The parasite showed morphological features distinct from all* Trypanosoma* species classified at that time. The flagellated form of the protozoan, similar to* Crithidia*, was found to colonize the posterior gut of hematophagous triatomines that infested the poorly built dwellings of villagers in Lasance in the northern region of the state of Minas Gerais in Brazil. After subjecting experimental apes to infected triatomines from the genus* Corynorhinus* spp., thus applying Koch's postulates, Chagas was able to identify a flagellated form in the bloodstream of the ape completely different from that found in insects. Chagas then associated the presence of the protozoan with the pathology observed in several residents from the region and began to study three supposedly infected children [57].

Microscopic visualization of the parasite allowed its identification as a Trypanosomatid based on recognition of the blepharoplast (now called kinetoplast) present in the different developmental forms of the parasite. Based on observations and previous knowledge obtained from other protozoan parasites, such as* Plasmodium *spp., Chagas classified more than ten different evolutionary stages of* T. cruzi* in fixed and stained samples [57]. In 1911 with the support of Carlos Chagas, Gaspar Vianna conducted extensive histological analyses of organs from infected experimental animals, which led him to simplify the classification of* T. cruzi* into two main evolutionary stages: a round-shaped form without an apparent flagellum (amastigote) and a slim flagellated form (trypomastigote) [58].

At that time, animals such as monkeys and dogs were used as experimental models for* in vivo* infections [57–60]. Because these were complex models and presented a challenge for visualizing intracellular parasites, investigation into* T. cruzi *biology was primarily based on microscopic observations of the peripheral blood from infected animals and patients. Simplification of experimental models from whole animals to* T. cruzi*-infected cell cultures* in vitro* was key to studying the* T. cruzi *life cycle and its developmental forms [61, 62]. Another important step was establishment of conditions to grow the parasite* in vitro. *This allowed a better understanding of the biology of the developmental forms found in vertebrate host cells and the invertebrate vector [63].

The first micrographic records of stained cells infected with* T. cruzi* were published in the 1930s and 1940s [59, 61], and the first microcinematographic record of the intracellular life cycle of the parasite was presented in the 1940s [64]. The pioneer recordings of Hertha Meyer by directly and continuously accessing parasites within single host mammalian cells confirmed the simplified model of the* T. cruzi *intracellular life cycle proposed by Vianna [58]. In collaboration with Keith Porter from Rockefeller University in the USA, Hertha Meyer was the first investigator to register the ultrastructure of* T. cruzi* invertebrate forms (epimastigotes) using electron microscopy [65]. Interestingly,* T. cruzi* was one of the first cells observed with this technique [66]. Current detailed knowledge of internal structures of different morphological stages of the parasite has been acquired based on comprehensive transmission electron microscopy (TEM) data and gradual improvement of the technique over the years [66]. Thus, based on these early studies, four main distinct evolutionary stages are assumed in* T. cruzi*: flagellated dividing forms (epimastigotes) found in the triatomine gut; infective slim flagellated forms (metacyclic trypomastigotes) at the rectal ampoule that, when released with the feces, may initiate host infection by infecting mammalian host cells; once free in the cytoplasm, they differentiate into multiplying intracellular round-shaped forms (amastigotes); after nine cycles of binary divisions [67], amastigotes differentiate into bloodstream trypomastigotes that burst out of infected cells, reach the circulation, and may infect other host cells or a triatomine in a future blood meal [63].

One of the first detailed time-lapse studies of the intracellular* T. cruzi* life cycle was performed in the early 1970s by Dvorak and Hyde [67]. Using microcinematographic recordings, they established a model that involves (i) an invasion (penetration) phase promoted by an infective flagellated form of the parasite; (ii) a first differentiation (reorganization) phase in which the flagellated forms turn into oval-shaped amastigote forms; (iii) a multiplication (reproduction) phase in which amastigotes multiply inside host cells; (iv) a second differentiation phase in which amastigotes differentiate back into flagellated forms; and (v) the last phase of the intracellular cycle (escape) in which the flagellated forms rupture the host cell and spread to the extracellular milieu [67]. “Continuous observations” by Dvorak and Hyde allowed a better understanding of parasite interactions with the host cell.

Possibly the most extensively studied aspect of the* T. cruzi *intracellular cycle is the internalization step, also referred to as penetration or invasion.* T. cruzi* infective forms, including metacyclic trypomastigotes (MTs), tissue culture trypomastigotes (TCTs; analogs to bloodstream trypomastigotes), and extracellular amastigotes (EAs), which are obtained by differentiating TCTs or bloodstream trypomastigotes* in vitro *and* in vivo,* respectively [68–72], invade host cells through distinct mechanisms that will be discussed in more detail.

In the late 1970s, Zanvil Cohn's group at Rockefeller University (1926–1993) showed that epimastigotes (noninfective forms) and MTs could be internalized by professional phagocytes and that only trypomastigotes could enter nonprofessional phagocytes via phagocytosis [73]. Additionally, the group observed that amastigotes released into cell culture supernatants could enter and multiply in all cell types examined. Infectivity of extracellular amastigotes was confirmed by others [69, 74–76]. Schenkman and colleagues later observed that MTs and TCTs preferentially entered polarized MDCK monolayers at the basolateral regions, whereas nonconfluent cell was mostly penetrated by TCTs at their borders [77]. Using subconfluent HeLa cells, Mortara (1991) [78] observed different patterns of parasite internalization when comparing MTs and EAs. In line with Schenkman's (1988) observations [77], MTs preferentially invaded at the edge of host cells; conversely, EAs initially bound and were then entangled by host cell microvilli at the dorsal surface of HeLa cells before internalization.

**Figure 2:**
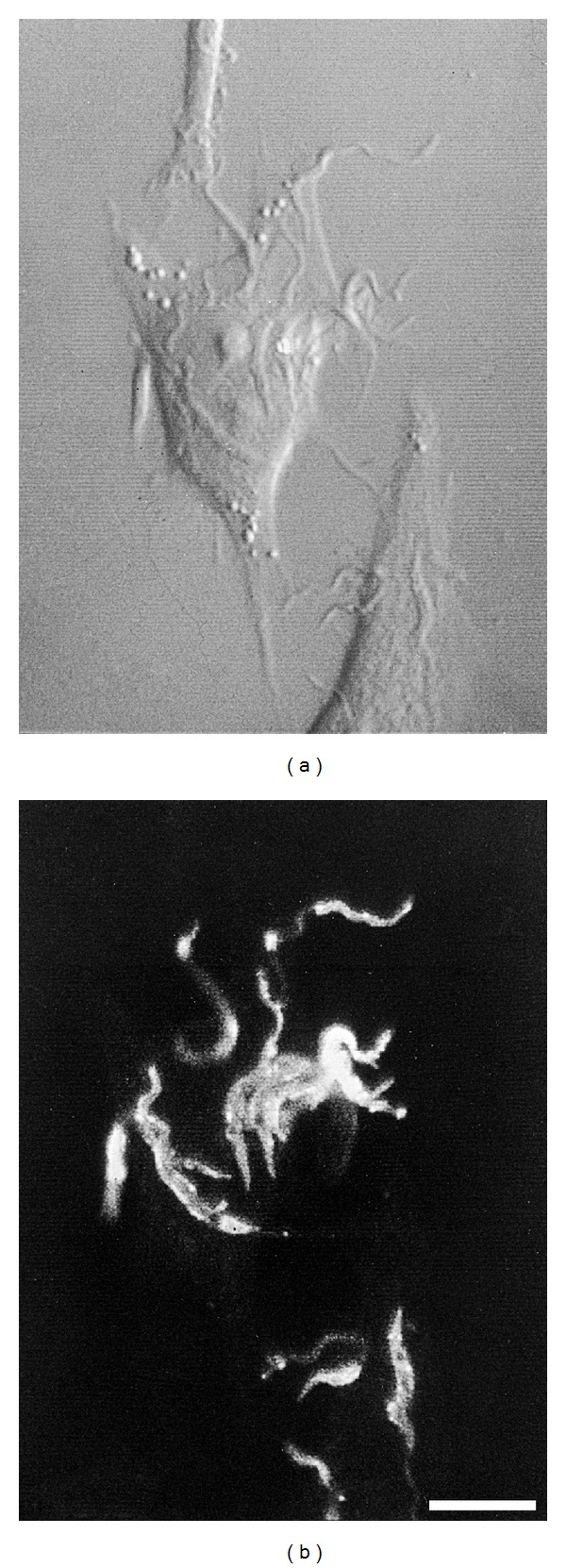
Cover of Memórias do Instituto Oswaldo Cruz, vol. 86 (1) [14]. Likely the first DIC image obtained with a confocal microscope (W. Brad Amos, personal communication) showing HeLa cell infection by G strain metacyclic trypomastigotes. On the right, the corresponding image after immunofluorescence with monoclonal antibody anti-35/50 kDa mucin, suggesting release of the molecule in parts of internalized parasites [15].

As immunofluorescence methodologies became popular in cell biology, they quickly grew to be valuable tools in studying* T. cruzi*-host cell interaction. Additionally, the advent of laser scanning confocal microscopy around the 1990s added significant improvements in both lateral and axial resolution on image acquisition compared to conventional wide field fluorescence. Protozoology also largely benefited from these techniques in that one of the first applications of confocal microscopy in studying the cell biology of parasitic infections was observation of actin redistribution in cells interacting with trypomastigotes [79]. Additionally, one of the first images combining Normarski DIC and confocal fluorescence imaging is of a HeLa cell interacting with metacyclic trypomastigotes immunostained with anti-mucin antibody 3F5 (W. Brad Amos, personal communication). The image shown in Figure 2 was that on the cover of a special issue of Memórias do Instituto Oswaldo Cruz [14].

*T. cruzi *developmental forms and their repertoire of distinct surface proteins trigger different signaling pathways that promote invasion. For example, MTs present an 82 kDa surface glycoprotein (GP-82) that is implicated in parasite internalization but does not trigger actin mobilization to invasion sites [80, 81]. So far, the involvement of host cell actin filaments in MTs and TCTs invasion remains controversial. Ferreira et al. observed that, during MTs host cell invasion, a surface glycoprotein GP-82 depolymerizes actin microfilaments while GP-35/50, another MTs surface molecule, induces actin recruitment [81]. Procópio and colleagues did not observe inhibitory effect of Cytochalasin D on host cell invasion of G strain MTs, concluding that actin filaments did not participate in MTs entry [80]. Regarding TCT invasion, contradictory results on involvement of host actin have also been described [79, 82–84].

**Figure 3:**
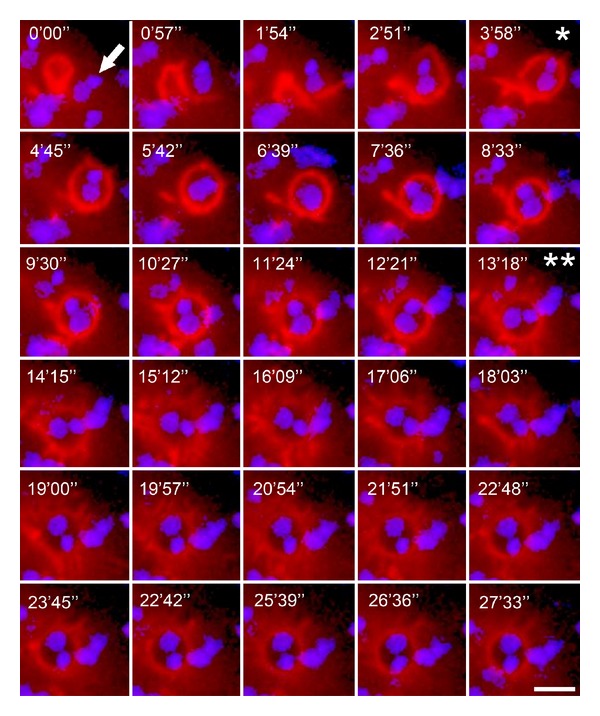
Actin recruitment by EAs in the phagocytic cup (Supplementary Video 2 available online at http://dx.doi.org/10.1155/2014/565291). HeLa cells transfected with fluorescent actin marker were incubated with EAs (arrow) and observed by time-lapse confocal microscopy (Leica SP5 TS) for 30 minutes at one frame per 57 seconds. Total EA internalization occurred within approximately 4 minutes (∗), but actin mobilization diffused approximately 9 minutes after total EA internalization (∗∗) 13 minutes after recording initiation. Actin is shown in red (Life-actin, ibidi); EA nucleus and kinetoplast are shown in blue (Hoescht 33258). Scale bar, 5 *μ*m.

By contrast, it is well established that EAs entry into host cells is highly dependent on actin mobilization [78]. EA invasion involves actin-rich cup-like structures that embrace the parasite, called the phagocytic cup (Figure 3 and supplementary Video 2) [85]. Fernandes and colleagues [86] recently demonstrated that EAs are able to trigger their own phagocytosis by HeLa cells. Using spinning-disk confocal microscopy, they observed that PVs formed by EAs remodeled their phosphoinositide content, which are important signaling components for subsequent fusion with other host cell vesicles. EA PVs first mature into a CD63-, followed by synaptotagmin VII- and then LAMP1-positive structures. These data show that EAs activate a phagocytic pathway in nonprofessional phagocytes that resembles large particle uptake by professional phagocytes [86].

Another application of immunofluorescence techniques in this area of research relates to the role of host cell lysosomes in* T. cruzi *invasion. Tardieux and colleagues [83] observed that lysosomes are recruited to TCT invasion sites, a process dependent on calcium that culminates with the formation of LAMP-positive* T. cruzi *PVs [87]. Norma Andrews' group (U. Maryland) demonstrated that TCTs induce plasma membrane lesions during the invasion process. These wounds are repaired by lysosomes that secrete sphingomyelinase, an enzyme that generates ceramide [88]. On the outer leaflet of the plasma membrane, this lipid induces inward budding that could drive parasite internalization. Using live imaging techniques, the authors confirmed previous TEM observations, showing the dynamics of lysosome mobilization towards cell periphery during interaction with trypomastigotes [89].

Based on the observation of PIP-3 recruitment by TCTs at early steps of interaction with mammalian cells, a lysosome-independent pathway for trypomastigote entry has also been described [90]. Although most of the results in this work consist of very compelling evidence, it is worth mentioning that Figure 2 (related to the attached supplementary video 1) clearly shows moving parasites from as early as 3 min (possibly under the cells). What is then referred to as the “second parasite” also appears moving in the field (possibly already inside the cell) and the so-called recruitment of Akt-PH-GFP for this parasite, that begins at around 13 minutes, is undoubtedly arising from the protrusion of the trypomastigote, actively moving from* inside* the cell. The implication of this observation is that these trypomastigotes most likely had invaded the imaged cell* before* this period. Considering the theme of this review, this might possibly be regarded as a misinterpretation of a rather compelling live image of* T. cruzi *trypomastigotes interacting with host cells. Recently, Barrias et al. [91] provided evidence suggesting that* T. cruzi* trypomastigotes may also subvert the macropinocytic pathway to enter host cells.

Interestingly, they also reported intracellular trypomastigotes protruding from within the host cell after 15 minutes of infection. Although the authors focused their observations on parasite entry, it appeared that parasites could also attempt to escape or egress from the host cell [89]. Similar behavior of internalized TCTs pushing out from infected cells had already been described by Dvorak and Hyde in their pioneering studies [67]. In 1992, Schenkman and Mortara [79] observed membrane protrusions and actin recruitment that were associated with TCT invasion sites in HeLa cells. At that time, fixed samples were visualized by confocal, transmission, and scanning electron microscopy (SEM). Static images were interpreted as depicting events associated with parasite entry. In light of observations made by Hyde and Dvorak and Fernandes et al. [16, 67], formation of pseudopodia described by Schenkman and Mortara [79] in fixed samples processed 30 minutes after cell invasion was most certainly related to protrusion of already-internalized parasites rather than internalization, as interpreted at the time. Integration of temporal information with spatial data invites careful contemplation of host-parasite interaction micrographs from fixed samples. In particular, considering* T. cruzi *trypomastigotes inside host cells and exposition of parasite flagella after host cell membrane damage [67, 89], static images published years ago could be ambiguously interpreted as both invasion and exit processes.

**Figure 4:**
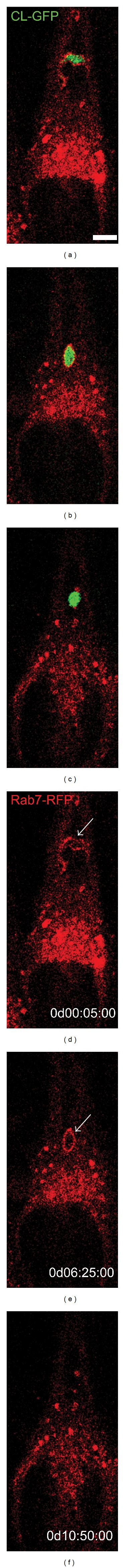
*T. cruzi* metacyclic trypomastigote forms begin to differentiate into amastigote-like forms inside the parasitophorous vacuole (supplementary Video 3). HeLa cells transfected with Rab7-Red fluorescent protein (RFP) were infected with metacyclic trypomastigotes (MTs) from a CL strain transfected with green fluorescent protein (GFP). Time-lapse images show the parasite internalized inside the parasitophorous vacuole (PV) labeled with Rab7-RFP (white arrow) after one hour. MTs differentiated into round-shaped forms, followed by loss of Rab-7 staining, suggesting parasite escape from the PV. Time-lapse acquisition is displayed as days : hours : minutes : seconds (dd : hh : mm : ss). Scale bar, 5 *μ*m. Images acquired with a confocal microscope (Leica SP5 TS).

After internalization, a poorly understood aspect of the* T. cruzi* intracellular life cycle is formation and escape from PVs. Ultrastructural studies demonstrated that, shortly after invasion (around 60 minutes),* T. cruzi *trypomastigotes are lodged in a vacuole surrounded by a thin membrane, and “at later times, all the parasites were seen free in the cytoplasm” [73]. This transient PV is able to fuse with host cell lysosomes in phagocytic and nonphagocytic cells, which is clearly observed by confocal and electron microscopy [73, 89, 90, 92–96]. The precise mechanisms by which parasites escape from PVs into the cell cytoplasm have not been fully disclosed, but* T. cruzi* trypomastigotes and amastigotes have been shown to secrete a membrane pore-forming protein, TC-TOX, which is active at pH 5.5 and could be implicated in PV rupture [97–99]. The question remains as to whether* T. cruzi* differentiates into amastigotes inside or outside the PV. de Carvalho and de Souza [95] suggested that trypomastigotes were able to disrupt PVs before differentiation into amastigotes, which is a feature of phagolysosomes in an acidic milieu. Indeed, it is possible to observe small pores in PV membranes that developed after 1 hour and 30 minutes of trypomastigotes infection in macrophages using TEM [95]. Using multidimensional live imaging of HeLa cells transfected with RFP-tagged Rab7 and infected with metacyclic forms of* T. cruzi* expressing GFP, we observed initial morphological changes of MT into round-shaped forms followed by dissolution of RFP-Rab7 around the parasite (Figure 4 and supplementary Video 3). In contrast to previous investigations, the data suggest that MT begins to differentiate into an amastigote form before escape to the host cell cytosol. Further experiments using multidimensional images and appropriate markers of* T. cruzi *differentiation will potentially reveal if differentiation into amastigotes takes place in PVs or in the cytosol and provide important information for future studies on drug delivery.

**Figure 5:**
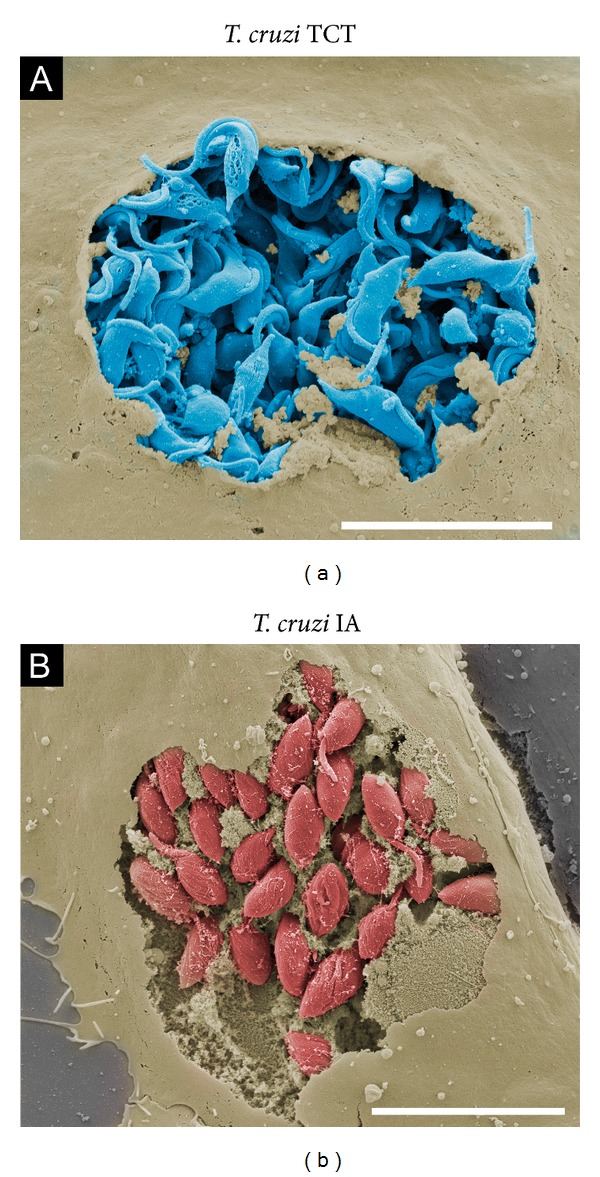
Visualization of the* T. cruzi* intracellular life cycle using field-emission scanning electron microscopy. (a) Tissue cultured trypomastigotes (TCTs) (blue) egress from Vero cells (light brown). (b) Intracellular amastigotes (red) of* T. cruzi* hosted by Vero cells (light brown). Infected cells were fixed with 4% paraformaldehyde and then subjected to electron scanning processing. Briefly, samples were dehydrated in an ethanol series, subjected to critical-point drying from CO_2_ and gold sputtering. In (b), samples processed as in (a) were fractured by adhesive tape and then gold sputtered. Scale bars, 10 *μ*m.

**Figure 6:**
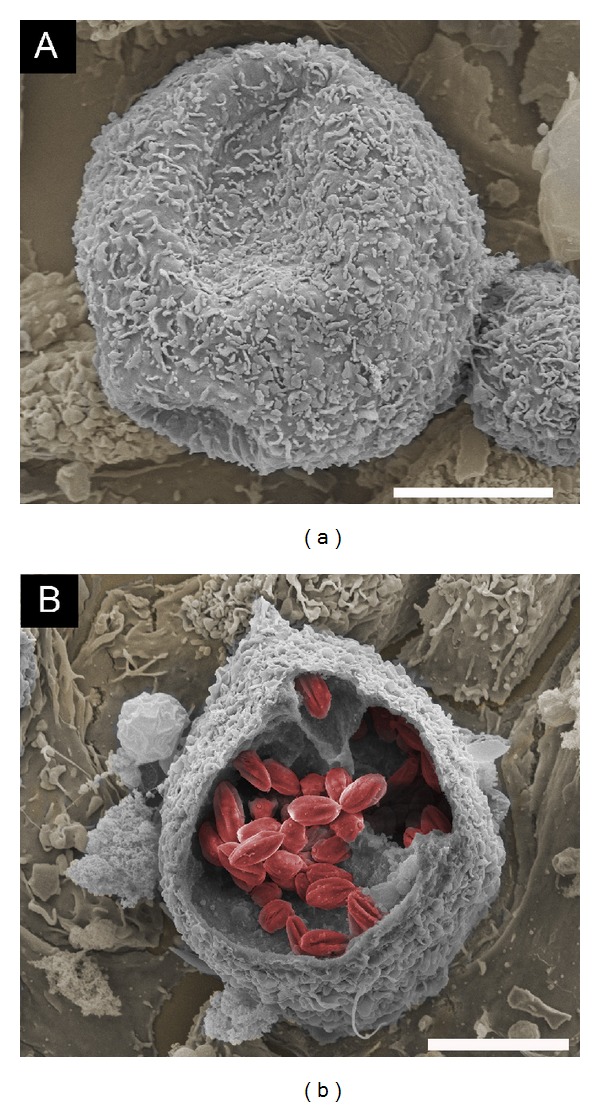
Visualization of bone marrow-derived macrophages infected with* L. amazonensis* using field-emission scanning electron microscopy. In (a), intact cell (light grey) and, in (b),* L. amazonensis* amastigotes (red) within spacious PVs were exposed through fracture by Scotch tape, followed by gold sputtering. The samples were processed as described in the legend of Figure 5. Scale bar, 10 *μ*m.

Egress from host cells is also poorly understood. Although host cell egress was highlighted in Hertha Meyer's recordings in the 1940s, there are few studies on the subject. Edgar Rowland's group was one of the first to systematically investigate* T. cruzi *egress using an interesting experimental approach: culture medium with serum obtained from chronically infected mice showed inhibition of parasite egress and a decrease in intracellular replication in fibroblasts [100, 101]. This inhibitory effect was also observed in serum obtained from chronic chagasic patients [102]. It is possible to hypothesize that antibodies (anti-egressins) are reaching intracellular parasites and, according to the authors, promoting intracellular agglutination of* T. cruzi *forms to block egress. At a later phase of the* T. cruzi* intracellular life cycle, the plasma membrane of infected host cells is weakened, leading to higher permeability to molecules, including antibodies [103].* T. cruzi *egress from host cells has also been investigated by our group. The precise moment of trypomastigote exit from a host cell was captured using field-emission scanning electron microscopy (FE-SEM) (Figure 5(a)). FE-SEM is a valuable microscopy tool to analyze biological surfaces with higher spatial resolution than SEM [104]. Various morphological and parasite-host cell interaction-related processes have been highlighted using conventional or FE-SEM, including the flagellar attachment zone [105], colonization forms in the insect vector and its excretion [106, 107], stimuli to differentiate its life cycle form and invasion [108–110], and cytoskeleton organization during infection [89, 111]. One of our aims using this technique was to try and observe intracellular parasites in host cells and entire organs using the ingenious “scotch tape technique,” which fractures the cell monolayer and tissue samples [112, 113]. This approach allowed us to observe intracellular amastigotes of* T. cruzi* in the cytoplasm of Vero cells (Figure 5(b)) as well as intracellular amastigotes of* L. amazonensis* located in large vacuoles of macrophages derived from mouse bone marrow (Figure 6(b)).

**Figure 7:**
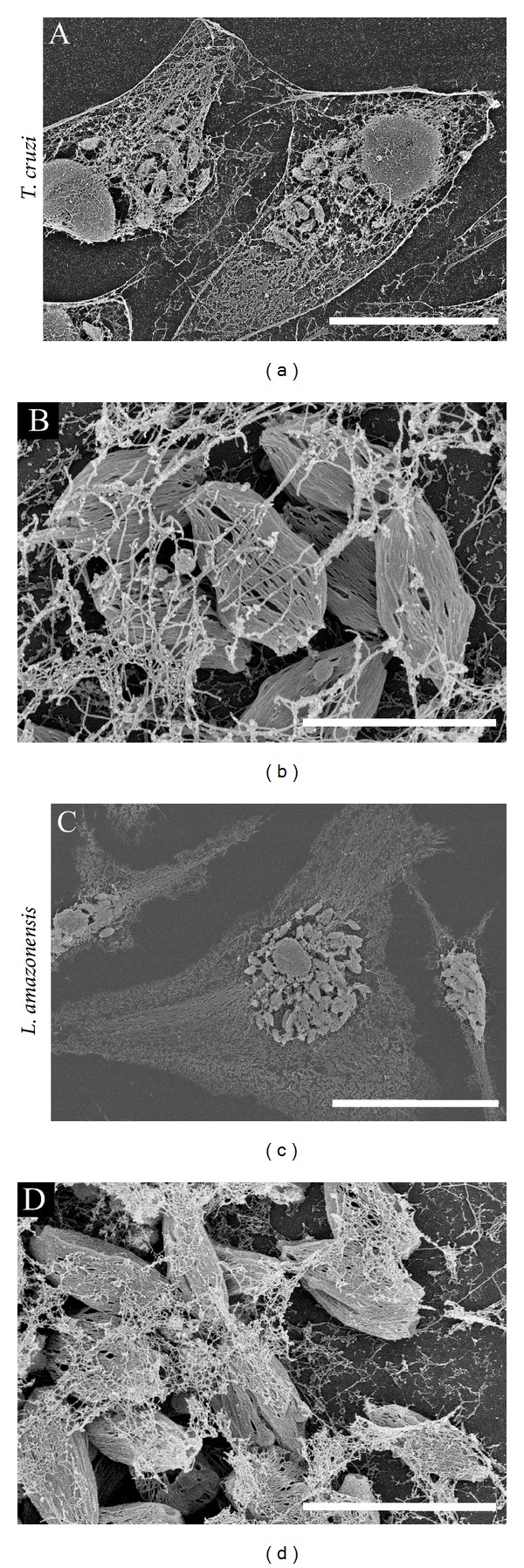
Visualization of host cell cytoskeleton networks and intracellular amastigotes of* T. cruzi *(a, b) and* L. amazonensis *(c, d) with host cell cytoskeleton networks. Infected HeLa cells (a, b) and mouse bone marrow macrophages (c, d) were treated with a membrane extraction solution containing Triton X-100, taxol, and phalloidin (to stabilize microtubules and microfilaments) [16, 17]. Cytoskeletons of infected cells were visualized by field emission scanning electron microscopy after processing and gold coating. Scale bars: (a) 20 *μ*m; (b) 3 *μ*m; (c) 30 *μ*m; (d) 5 *μ*m.

Several protocols have been used to visualize host cytoskeleton interaction with parasites using EM. Fernandes and colleagues [89] treated infected cells with a membrane extraction solution containing Triton X-100, taxol, and phalloidin to stabilize microtubules and microfilaments [17]. This strategy enabled the authors to visualize the initial invasion profile using TEM (to generate a three-dimensional projection) in which the posterior end of trypomastigotes penetrates underneath HeLa cells, resulting in actin filament enrichment at the undulated cell cortex [86]. We used the same approach to visualize intracellular amastigotes in the host cell cytoplasm. As shown in Figure 7, we observed intracellular amastigotes of* T. cruzi* (Figure 7(a)) and* L. amazonensis *(Figure 7(b)) hosted by cells in which the cytoskeleton network was preserved. In these images, amastigotes were also subjected to membrane extraction to observe internal structures of the parasites.

**Figure 8:**
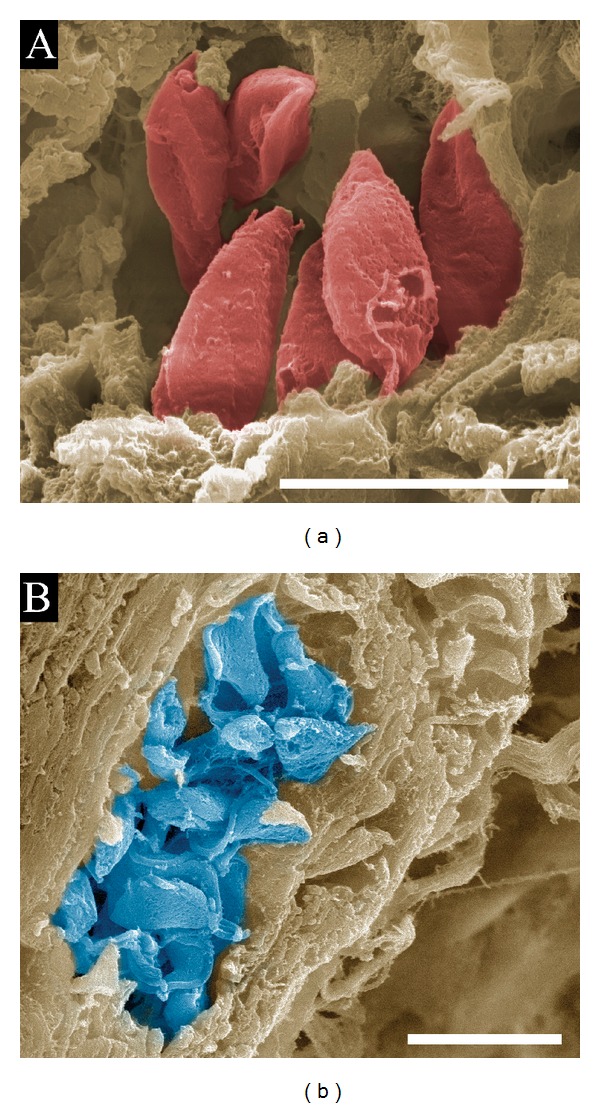
Field-emission scanning electron microscopy of mouse hearts infected with* T. cruzi.* Thick paraffin embedded sections of mouse hearts infected with Y strain metacyclic trypomastigotes were deparaffinized and processed for field emission scanning electron microscopy [18–21]. Briefly, paraffin was removed by melting the sections block and then deparaffinized with xylol and ethanol. Next, heart muscle sections cut with a razor blade were dehydrated in an ethanol series, subjected to critical-point drying, and gold sputtered. (a) Amastigotes (red), scale bar, 4 *μ*m; (b) trypomastigotes (blue), scale bar, 10 *μ*m.

Our group has focused efforts on the observation of intracellular parasites in infected hearts of mice at the SEM level. Detailed information from infected cardiac tissue is relevant for elucidating* T. cruzi *pathogenesis due to heart tissue damage caused by the parasite and/or autoimmune effects, which are poorly understood and controversial [114]. Pathological investigations on fatal cases of Chagas disease performed by Gaspar Vianna in association with the German pathologist Hermann Dürk in 1917 defined acute and chronic phases of the disease, with the latter phase associated with cardiac involvement [115]. The association between* T. cruzi *infection and cardiac failure in chronic patients was a well-established concept by the 1960s [116]. Common techniques for SEM visualization of internal structures, such as cryofracturing, freeze fracturing or microdissection, either are not precise enough for observing localized histological events or require specialized trained personnel in addition to high financial and equipment costs necessary to perform these procedures. Other researchers have performed SEM in paraffin-histological sections within their respective fields of research [18–21], but an image of* T. cruzi*-infected tissue from thick sections (>40 *μ*m) has not been produced. In thick paraffin histological sections submitted for SEM processing, we observed* T. cruzi* amastigote and trypomastigote nests within heart muscle fibers (Figures 8(a) and 8(b)). This simple, cost-effective, and rapid approach was applied after conventional formaldehyde fixation and paraffin embedding, followed by deparaffination with xylol, dehydration with ethanol, critical-point drying, and sputter-coating with gold for SEM. Mice hearts were stored in paraffin blocks for several years before they were processed using SEM, highlighting the good condition of the tissue and its structures despite a long period of time in storage. A related and relevant issue that deserves more in-depth study is understanding how circulating parasites reach this organ. Intravital imaging techniques of whole animals and multiphoton confocal microscopy of infected tissues should allow for fluorescent-tagged* T. cruzi *tracking in what could become a challenging and encouraging perspective for future investigations.

## 4. Concluding Remarks

Innovative techniques consistently improve our interpretations of biological processes and their mechanisms in biomedical research. In this review, we presented examples of advances in microscopy that contributed to building concepts regarding host-parasite interactions of the human kinetoplastid parasites* Leishmania* spp. and* T. cruzi*. There are several other cases of conceptual breakthroughs that we did not cover in this review on microscopy, including newly developed techniques that could certainly lead to important changes in how we conceptualize similar intracellular parasites. Namely, electron tomography in cryopreserved samples allows for 3D reconstruction of infected cells and parasites bypassing cumbersome serial slicing; superresolution microscopes (PALM/STORM and STED) increase optical resolution to tens of nanometers and allow for live imaging; bioluminescent parasites could be tracked in whole organisms using* in vivo* bioluminescent imaging systems [117, 118]; and use of reporters, probes, or other microcopy techniques (FRAP, FRET and FLIM) improves microscopic observations regarding biochemical/molecular mechanisms of host/pathogen interactions. We can rely on history to repeat itself in that further studies using these cutting-edge microscopic technologies will change our perception of* Leishmania *spp. and* T. cruzi *intracellular parasitism and contribute to the development of novel and more efficient strategies of chemotherapy and vaccination.

## Supplementary Material

Supplementary Material includes three Supplementary Videos: The first video presents *Leishmania amazonensis* amastigotes cell to cell transfer (Supplementary Video 1). The Supplementary Video 2 demonstrates actin recruitment by *Trypanosoma cruzi* extracellular amastigotes in live HeLa cells. In Supplementary Video 3 it is observed that *Trypanosoma cruzi* metacyclic forms differentiate into round-shaped forms and lose Rab-7 marker in transfected HeLa cells.
Video 1: Cinematographic recordings performed by P. Veras, M. Pouchelet and M. Rabinovitch in 1994. The video shows two situations in which *L. amazonensis* amastigotes were transferred from cell to cell: from lymphocyte to macrophage in which lymphocytes behave similar to a Trojan Horse; and from macrophage to macrophage in which an intact amastigote appeared to be transferred without phagocytosis of the whole infected cell.
Video 2: Actin recruitment by *T. cruzi* extracellular amastigotes in live HeLa cells. HeLa cells transfected with fluorescent actin marker (LifeAct, ibidi) were incubated with *T. cruzi* extracellular amastigotes (EAs) and observed by time-lapse confocal microscopy for 30 minutes, at one frame per 57 seconds. In this interaction, total EA internalization occurred within approximately 4 minutes, but actin mobilization (red) diffused approximately after only 9 minutes total of EA internalization. Nuclei and kinetoplasts were labeled with Hoechst 33342 (blue). Bar = 5 µm.
Video 3: After invading HeLa cells (~3h), *T. cruzi* metacyclic trypomastigote forms differentiate into round-shaped forms and lose Rab-7 (early endosomal marker). HeLa cells transfected with Rab7-red fluorescent protein (RFP) (red) were incubated with *T. cruzi* metacyclic forms transfected with green fluorescent protein (GFP) (green) and observed by time-lapse confocal microscopy. The parasite was inside the parasitophorous vacuole (PV) with the Rab7-RFP marker. Initially, metacyclic *T. cruzi* differentiates into a round-shaped form and later (between 9h:35min and 9h:50min) the Rab7-RFP marker is lost, suggesting parasite escape from the PV.

## Figures and Tables

**Box 1:**
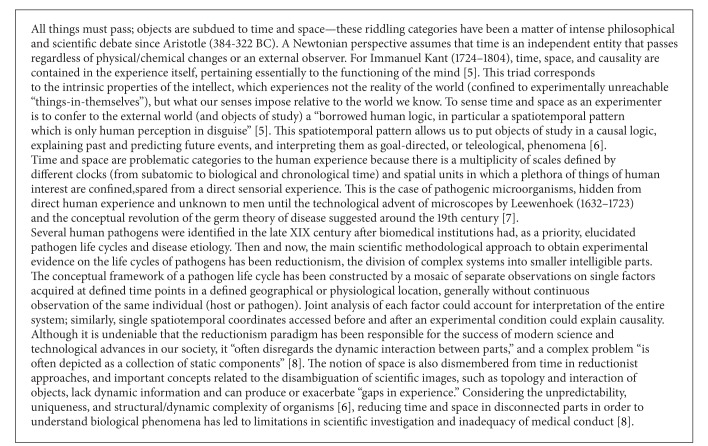
A Philosophical Introduction to the Unobservable.

## References

[1] Moreira D, López-García P, Vickerman K (2004). An updated view of kinetoplastid phylogeny using environmental sequences and a closer outgroup: proposal for a new classification of the class Kinetoplastea. *International Journal of Systematic and Evolutionary Microbiology*.

[2] Stuart K, Brun R, Croft S (2008). Kinetoplastids: related protozoan pathogens, different diseases. *Journal of Clinical Investigation*.

[3] de Souza W, de Carvalho TMU, Barrias ES (2010). Review on *Trypanosoma cruzi*: host cell interaction. *International Journal of Cell Biology*.

[4] Rosenberger R (2011). A case study in the applied philosophy of imaging: the synaptic vesicle debate. *Science Technology and Human Values*.

[5] Boxenbaum H (1986). Time concepts in physics, biology, and pharmacokinetics. *Journal of Pharmaceutical Sciences*.

[6] Mayr E (1961). Cause and effect in biology—kinds of causes, predictability, and teleology are viewed by a practicing biologist. *Science*.

[7] Egaña Aranguren M, Wolstencroft K, Sattler U (2007). Using OWL to model biological knowledge. *International Journal of Human Computer Studies*.

[8] Ahn AC, Tewari M, Poon C-S, Phillips RS (2006). The limits of reductionism in medicine: could systems biology offer an alternative?. *PLoS Medicine*.

[9] Sattler R (1986). *Biophilosophy: Analytic and Holistic Perspectives*.

[10] (2009). Milestones in light microscopy. *Nature Cell Biology*.

[11] Xiao GQ, Corle TR, Kino GS (1988). Real-time confocal scanning optical microscope. *Applied Physics Letters*.

[12] Landecker H (2009). Seeing things: from microcinematography to live cell imaging. *Nature Methods*.

[13] Lofgren R (1950). The structure of *Leishmania* tropica as revealed by phase and electron microscopy. *Journal of Bacteriology*.

[14] Mortara RA (1991). Differential interference contrast and confocal fluorescence photomicrograph showing the distribution of the 35/50 kDa surface glycoconjugate of *Trypanosoma cruzi* trypomastigotes during invasion of HeLa cells. *Memórias do Instituto Oswaldo Cruz*.

[15] Schenkman S, Ferguson MAJ, Heise N, Cardoso de Almeida ML, Mortara RA, Yoshida N (1993). Mucin-like glycoproteins linked to the membrane by glycosylphosphatidylinositol anchor are the major acceptors of sialic acid in a reaction catalyzed by trans-sialidase in metacyclic forms of *Trypanosoma cruzi*. *Molecular and Biochemical Parasitology*.

[16] Fernandes MC, de Andrade LR, Andrews NW, Mortara RA (2011). *Trypanosoma cruzi* trypomastigotes induce cytoskeleton modifications during hela cell invasion. *Memórias do Instituto Oswaldo Cruz*.

[17] Sant’Anna C, Campanati L, Gadelha C (2005). Improvement on the visualization of cytoskeletal structures of protozoan parasites using high-resolution field emission scanning electron microscopy (FESEM). *Histochemistry and Cell Biology*.

[18] Gaudet DA, Kokko EG (1984). Application of scanning electron microscopy to paraffin-embedded plant tissues to study invasive process of plant-pathogenic fungi. *Phytopathology*.

[19] Geissinger HD (1971). Correlated light optical and scanning electron microscopy of Gram smears of bacteria and paraffin sections of cardiac muscle. *Journal of Microscopy*.

[20] Wergin WP, Yaklich RW, Roy S (1997). Imaging thin and thick sections of biological tissue with the secondary electron detector in a field-emission scanning electron microscope. *Scanning*.

[21] Russell SD, Daghlian CP (1985). Scanning electron microscopic observations on deembedded biological tissue sections: comparison of different fixatives and embedding materials. *Journal of Electron Microscopy Technique*.

[22] Lainson R, Shaw JJ (1987). *Evolution, Classification and Geographical Distribution*.

[23] Altamirano-Enciso AJ, Marzochi MCA, Moreira JS, Schubach AO, Marzochi KBF (2003). On the origin and spread of cutaneous and mucosal leishmaniasis, based on pre- and post- colombian historical source. *História Ciências Saúde, Manguinhos,*.

[24] Dedet J-P (2007). Edmond Sergent’s discoveries on the vectorial transmission of agents of human and animal infectious diseases. *Bulletin de la Societé de Pathologie Exotique*.

[25] Théodoridès J (1997). Historical note on the discovery of the transmission of cutaneous leishmaniasis by phlebotomes. *Bulletin de la Societé de Pathologie Exotique*.

[26] Wright JH (1903). Protozoa in a case of tropical ulcer (“Delhi Sore”). *Journal of Medical Research*.

[27] Christophers SR (1904). On a parasite found in persons suffering from enlargement of the spleen in India. *Second Report*.

[28] Heyneman D (1971). Immunology of leishmaniasis. *Bulletin of the World Health Organization*.

[29] Ueno N, Wilson ME (2012). Receptor-mediated phagocytosis of *Leishmania*: implications for intracellular survival. *Trends in Parasitology*.

[30] Pulvertaft RJV, Hoyle GF (1960). Stages in the life-cycle of *Leishmania donovani*. *Transactions of the Royal Society of Tropical Medicine and Hygiene*.

[31] Miller HC, Twohy DW (1967). Infection of macrophages in culture by leptomonads of *Leishmania donovani*. *The Journal of Protozoology*.

[32] Akiyama HJ, Haight RD (1971). Interaction of *Leishmania donovani* and hamster peritoneal macrophages. A phase-contrast microscopical study. *American Journal of Tropical Medicine and Hygiene*.

[33] Forestier C-L, MacHu C, Loussert C, Pescher P, Späth GF (2011). Imaging host cell-*Leishmania* interaction dynamics implicates parasite motility, lysosome recruitment, and host cell wounding in the infection process. *Cell Host and Microbe*.

[34] Courret N, Fréhel C, Gouhier N (2002). Biogenesis of *Leishmania*-harbouring parasitophorus vacuoles following phagocytosis of the metacyclic promastigote or amastigote stages of the parasites. *Journal of Cell Science*.

[35] Aikawa M, Hendricks LD, Ito Y, Jagusiak M (1982). Interactions between macrophagelike cells and *Leishmania braziliensis in vitro*. *American Journal of Pathology*.

[36] Blackwell JM, Plant JE (1986). Expression of the natural resistance gene (Lsh) in wild mice infected experimentally with *Leishmania donovani* or *Salmonella typhimurium*. *Current topics in Microbiology and Immunology*.

[37] Love DC, Kane MM, Mosser DM (1998). *Leishmania amazonensis*: the phagocytosis of amastigotes by macrophages. *Experimental Parasitology*.

[38] Coling D, Kachar B (2001). Theory and application of fluorescence microscopy. *Current Protocols in Neuroscience*.

[39] Lodge R, Descoteaux A (2005). *Leishmania donovani* promastigotes induce periphagosomal F-actin accumulation through retention of the GTPase Cdc42. *Cellular Microbiology*.

[40] Lodge R, Descoteaux A (2006). Phagocytosis of *Leishmania donovani* amastigotes is Rac1 dependent and occurs in the absence of NADPH oxidase activation. *European Journal of Immunology*.

[41] Alexander J, Vickerman K (1975). Fusion of host cell secondary lysosomes with the parasitophorous vacuoles of *Leishmania mexicana* infected macrophages. *The Journal of Protozoology*.

[42] Chang KP, Dwyer DM (1978). *Leishmania donovani*. Hamster macrophage interactions *in vitro*: cell entry, intracellular survival, and multiplication of amastigotes. *Journal of Experimental Medicine*.

[43] Antoine J-C, Prina E, Lang T, Courret N (1998). The biogenesis and properties of the parasitophorous vacuoles that harbour *Leishmania* in murine macrophages. *Trends in Microbiology*.

[44] Prina E, Antoine Cl. J (1990). Localization and activity of various lysosomal proteases in rat macrophages infected with *Leishmania amazonensis*. *Pathologie Biologie*.

[45] Antoine J-C, Prina E, Jouanne C, Bongrand P (1990). Parasitophorous vacuoles of *Leishmania amazonensis*-infected macrophages maintain an acidic pH. *Infection and Immunity*.

[46] Lang T, de Chastellier C, Frehel C (1994). Distribution of MHC class I and of MHC class II molecules in macrophages infected with *Leishmania amazonensis*. *Journal of Cell Science*.

[47] Handman E, Bullen DV (2002). Interaction of *Leishmania* with the host macrophage. *Trends in Parasitology*.

[48] Real F, Mortara RA (2012). The diverse and dynamic nature of *Leishmania* parasitophorous vacuoles studied by multidimensional imaging. *PLoS Neglected Tropical Diseases*.

[49] Lang T, Lecoeur H, Prina E (2009). Imaging *Leishmania* development in their host cells. *Trends in Parasitology*.

[50] Lippuner C, Paape D, Paterou A (2009). Real-time imaging of *Leishmania mexicana*-infected early phagosomes: a study using primary macrophages generated from green fluorescent protein-Rab5 transgenic mice. *FASEB Journal*.

[51] Ridley DS (1980). A histological classification of cutaneous leishmaniasis and its geographical expression. *Transactions of the Royal Society of Tropical Medicine and Hygiene*.

[52] Rittig MG, Schröppel K, Seack K-H (1998). Coiling phagocytosis of trypanosomatids and fungal cells. *Infection and Immunity*.

[53] Rittig MG, Bogdan C (2000). *Leishmania*-host-cell interaction: complexities and alternative views. *Parasitology Today*.

[54] Peters NC, Egen JG, Secundino N (2008). In vivo imaging reveals an essential role for neutrophils in leishmaniasis transmitted by sand flies. *Science*.

[55] Ng LG, Hsu A, Mandell MA (2008). Migratory dermal dendritic cells act as rapid sensors of protozoan parasites. *PLoS Pathogens*.

[56] Gueirard P, Laplante A, Rondeau C, Milon G, Desjardins M (2008). Trafficking of *Leishmania donovani* promastigotes in non-lytic compartments in neutrophils enables the subsequent transfer of parasites to macrophages. *Cellular Microbiology*.

[57] Chagas C (1909). Nova tripanozomiaze humana: estudos sobre a morfolojia e o ciclo evolutivo do*Schizotrypanum cruzi* n. gen., n. sp., ajente etiolojico de nova entidade morbida do homem. *Memórias do Instituto Oswaldo Cruz*.

[58] Vianna G (1911). Contribuição para o estudo da anatomia patolójica da “Moléstia de Carlos Chagas” (Esquizotripanose humana ou tireoidite parasitária). *Memórias do Instituto Oswaldo Cruz*.

[59] Dias E (1934). Estudos sobre o *Schizotrypanum cruzi*. *Memórias Do Instituto Oswaldo Cruz*.

[60] Villela E, Torres CM (1926). Histopathology of the central nervous system in experimental paralysis caused by *Schizotrypanum cruzi*. *Memórias do Instituto Oswaldo Cruz*.

[61] Romaña C, Meyer H (1942). Estudo do ciclo evolutivo do, “*Schizotrypanum cruzi*” em cultura de tecidos de embrião de galinha. *Memórias do Instituto Oswaldo Cruz*.

[62] Kofoid CA, Wood FD, McNeil ECE (1935). *The Cycle of Trypanosoma Cruzi in Tissue Culture of Embryonic Heart Muscle*.

[63] Brener Z (1973). Biology of *Trypanosoma cruzi*. *Annual Review of Microbiology*.

[64] Meyer H, Barasa A Life cycle of *Schizotrypanum cruzi* in tissue cultures. ,http://www.itarget.com.br/newclients/sbpz.org.br/2011/extra/download/cruzi1.mpg.

[65] Meyer H, Porter KR (1954). A study of *Trypanosoma cruzi* with the electron microscope. *Parasitology*.

[66] de Souza W (2008). Electron microscopy of trypanosomes—a historical view. *Memórias do Instituto Oswaldo Cruz*.

[67] Dvorak JA, Hyde TP (1973). *Trypanosoma cruzi*: interaction with vertebrate cells *in vitro*. I. Individual interactions at the cellular and subcellular levels. *Experimental Parasitology*.

[68] Ferreira ER, Bonfim-Melo A, Mortara RA, Bahia D (2012). *Trypanosoma cruzi* extracellular amastigotes and host cell signaling: more pieces to the puzzle. *Frontiers in Immunology*.

[69] Ley V, Andrews NW, Robbins ES, Nussenzweig V (1988). Amastigotes of *Trypanosoma cruzi* sustain an infective cycle in mammalian cells. *Journal of Experimental Medicine*.

[70] Lima FM, Oliveira P, Mortara RA, Silveira JF, Bahia D (2010). The challenge of Chagas’ disease: has the human pathogen, *Trypanosoma cruzi*, learned how to modulate signaling events to subvert host cells?. *New Biotechnology*.

[71] Mortara RA, Andreoli WK, Tantwaki NN (2005). Mammalian cell invasion and intracellular trafficking by *Trypanosoma cruzi* infective forms. *Anais da Academia Brasileira de Ciencias*.

[72] Tomlinson S, Vandekerckhove F, Frevert U, Nussenzweig V (1995). The induction of *Trypanosoma cruzi* trypomastigote to amastigote transformation by low pH. *Parasitology*.

[73] Nogueira N, Cohn Z (1976). *Trypanosoma cruzi*: mechanism of entry and intracellular fate in mammalian cells. *Journal of Experimental Medicine*.

[74] Behbehani K (1973). Developmental cycles of *Trypanosoma (Schizotrypanum) cruzi* (Chagas, 1909) in mouse peritoneal macrophages *in vitro*. *Parasitology*.

[75] Hudson L, Snary D, Morgan SJ (1984). *Trypanosoma cruzi*: continuous cultivation with murine cell lines. *Parasitology*.

[76] Chia-Tung Pan S (1978). *Trypanosoma cruzi*: *in vitro* interactions between cultured amastigotes and human skin-muscle cells. *Experimental Parasitology*.

[77] Schenkman S, Andrews NW, Nussenzweig V, Robbins ES (1988). *Trypanosoma cruzi* invade a mammalian epithelial cell in a polarized manner. *Cell*.

[78] Mortara RA (1991). *Trypanosoma cruzi*: amastigotes and trypomastigotes interact with different structures on the surface of HeLa cells. *Experimental Parasitology*.

[79] Schenkman S, Mortara RA (1992). HeLa cells extend and internalize pseudopodia during active invasion by *Trypanosoma cruzi* trypomastigotes. *Journal of Cell Science*.

[80] Procópio DO, da Silva S, Cunningham CC, Mortara RA (1998). *Trypanosoma cruzi*: effect of protein kinase inhibitors and cytoskeletal protein organization and expression on host cell invasion by amastigotes and metacyclic trypomastigotes. *Experimental Parasitology*.

[81] Ferreira D, Cortez M, Atayde VD, Yoshida N (2006). Actin cytoskeleton-dependent and -independent host cell invasion by *Trypanosoma cruzi* is mediated by distinct parasite surface molecules. *Infection and Immunity*.

[82] Schenkman S, Robbins ES, Nussenzweig V (1991). Attachment of *Trypanosoma cruzi* to mammalian cells requires parasite energy, and invasion can be independent of the target cell cytoskeleton. *Infection and Immunity*.

[83] Tardieux I, Webster P, Ravesloot J (1992). Lysosome recruitment and fusion are early events required for trypanosome invasion of mammalian cells. *Cell*.

[84] Fonseca Rosestolato CT, da Matta Furniel Dutra J, de Souza W, Ulisses de Carvalho TM (2002). Participation of host cell actin filaments during interaction of trypomastigote forms of *Trypanosoma cruzi* with host cells. *Cell Structure and Function*.

[85] Procópio DO, Barros HC, Mortara RA (1999). Actin-rich structures formed during the invasion of cultured cells by infective forms of *Trypanosoma cruzi*. *European Journal of Cell Biology*.

[86] Fernandes MC, Flannery AR, Andrews N, Mortara RA (2013). Extracellular amastigotes of *Trypanosoma cruzi* are potent inducers of phagocytosis in mammalian cells. *Cellular Microbiology*.

[87] Burleigh BA, Woolsey AM (2002). Cell signalling and *Trypanosoma cruzi* invasion. *Cellular Microbiology*.

[88] Tam C, Idone V, Devlin C (2010). Exocytosis of acid sphingomyelinase by wounded cells promotes endocytosis and plasma membrane repair. *Journal of Cell Biology*.

[89] Fernandes MC, Cortez M, Flannery AR, Tam C, Mortara RA, Andrews NW (2011). *Trypanosoma cruzi* subverts the sphingomyelinase-mediated plasma membrane repair pathway for cell invasion. *Journal of Experimental Medicine*.

[90] Woolsey AM, Sunwoo L, Petersen CA, Brachmann SM, Cantley LC, Burleigh BA (2003). Novel Pl 3-kinase-dependent mechanisms of trypanosome invasion and vacuole maturation. *Journal of Cell Science*.

[91] Barrias ES, Reignault LC, de Souza W, Carvalho TM (2012). *Trypanosoma cruzi* uses macropinocytosis as an additional entry pathway into mammalian host cell. *Microbes and Infection*.

[92] Andrade LO, Andrews NW (2004). Lysosomal fusion is essential for the retention of *Trypanosoma cruzi* inside host cells. *Journal of Experimental Medicine*.

[93] Caradonna KL, Burleigh BA (2011). Mechanisms of host cell invasion by *Trypanosoma cruzi*. *Advances in Parasitology*.

[94] Tanowitz H, Wittner M, Kress Y, Bloom B (1975). Studies of *in vitro* infection by *Trypanosoma cruzi*. I. Ultrastructural studies on the invasion of macrophages and L cells. *American Journal of Tropical Medicine and Hygiene*.

[95] Ulisses de Carvalho TM, de Souza W (1989). Early events related with the behaviour of *Trypanosoma cruzi* within an endocytic vacuole in mouse peritoneal macrophages. *Cell Structure and Function*.

[96] de Araújo-Jorge TC (1989). The biology of *Trypanosoma cruzi*-macrophage interaction. *Memórias do Instituto Oswaldo Cruz*.

[97] Rubin-de-Celis SSC, Uemura H, Yoshida N, Schenkman S (2006). Expression of trypomastigote trans-sialidase in metacyclic forms of *Trypanosoma cruzi* increases parasite escape from its parasitophorous vacuole. *Cellular Microbiology*.

[98] Andrews NW, Whitlow MB (1989). Secretion of *Trypanosoma cruzi* of a hemolysin active at low pH. *Molecular and Biochemical Parasitology*.

[99] Andrews NW, Abrams CK, Slatin SL, Griffiths G (1990). A *T. cruzi*-secreted protein immunologically related to the complement component C9: evidence for membrane pore-forming activity at low pH. *Cell*.

[100] Moore-Lai D, Rowland E (2004). Discovery and characterization of an antibody, anti-egressin, that is able to inhibit *Trypanosoma cruzi* egress *in vitro*. *Journal of Parasitology*.

[101] Wendelken JL, Rowland EC (2009). Agglutination of *Trypanosoma cruzi* in infected cells treated with serum from chronically infected mice. *Journal of Parasitology*.

[102] Costales J, Rowland EC (2005). Human chagasic serum contains antibodies capable of inhibiting *Trypanosoma cruzi* egress from tissue culture cells. *Journal of Parasitology*.

[103] Costales J, Rowland EC (2007). A role for protease activity and host-cell permeability during the process of *Trypanosoma cruzi* egress from infected cells. *Journal of Parasitology*.

[104] Coene W, Janssen G, Op de Beeck M, van Dyck D (1992). Phase retrieval through focus variation for ultra-resolution in field-emission transmission electron microscopy. *Physical Review Letters*.

[105] Rocha GM, Brandão BA, Mortara RA, Attias M, de Souza W, Carvalho TMU (2006). The flagellar attachment zone of *Trypanosoma cruzi* epimastigote forms. *Journal of Structural Biology*.

[106] Zeledón R, Bolaños R, Espejo Navarro MR, Rojas M (1988). Morphological evidence by scanning electron microscopy of excretion of metacyclic forms of *Trypanosoma cruzi* in vector’s urine. *Memorias do Instituto Oswaldo Cruz*.

[107] Boker CA, Schaub GA (1984). Scanning electron microscopic studies of *Trypanosoma cruzi* in the rectum of its vector *Triatoma infestans*. *Zeitschrift fur Parasitenkunde*.

[108] Bonaldo MC, Souto-Padron T, de Souza W, Goldenberg S (1988). Cell-substrate adhesion during *Trypanosoma cruzi* differentiation. *Journal of Cell Biology*.

[109] Barrias ES, Dutra JMF, de Souza W, Carvalho TMU (2007). Participation of macrophage membrane rafts in *Trypanosoma cruzi* invasion process. *Biochemical and Biophysical Research Communications*.

[110] Mortara RA, Minelli LMS, Vandekerckhove F, Nussenzweig V, Juarez Ramalho-Pinto F (2001). Phosphatidylinositol-specific phospholipase C (PI-PLC) cleavage of GPI-anchored surface molecules of *Trypanosoma cruzi* triggers *in vitro* morphological reorganization of trypomastigotes. *Journal of Eukaryotic Microbiology*.

[111] Rocha GM, Miranda K, Weissmüller G, Bisch PM, de Souza W (2008). Ultrastructure of *Trypanosoma cruzi* revisited by atomic force microscopy. *Microscopy Research and Technique*.

[112] Magno RC, Lemgruber L, Vommaro RC, de Souza W, Attias M (2005). Intravacuolar network may act as a mechanical support for *Toxoplasma gondii* inside the parasitophorous vacuole. *Microscopy Research and Technique*.

[113] Flood PR (1975). Dry-fracturing techniques for the study of soft internal biological tissues in the scanning electron microscope. *Scanning Electron Microscopy*.

[114] Cox FEG (2002). History of human parasitology. *Clinical Microbiology Reviews*.

[115] Perleth M (1997). The discovery of Chagas’ disease and the formation of the early Chagas’ disease concept. *History and philosophy of the life sciences*.

[116] Köberle F (1968). Chagas’ disease and chagas’ syndromes: the pathology of American trypanosomiasis. *Advances in Parasitology*.

[117] de la Llave E, Lecoeur H, Besse A, Milon G, Prina E, Lang T (2011). A combined luciferase imaging and reverse transcription polymerase chain reaction assay for the study of *Leishmania* amastigote burden and correlated mouse tissue transcript fluctuations. *Cellular Microbiology*.

[118] Goyard S, Dutra PL, Deolindo P, Autheman D, D'Archivio S, Minoprio P (2013). *In vivo* imaging of trypanosomes for a better assessment of host-parasite relationships and drug efficacy. *Parasitology International*.

